# Contribution of Mitochondrial Reactive Oxygen Species to Chronic Hypoxia-Induced Pulmonary Hypertension

**DOI:** 10.3390/antiox12122060

**Published:** 2023-11-30

**Authors:** Simin Yan, Joshua R. Sheak, Benjimen R. Walker, Nikki L. Jernigan, Thomas C. Resta

**Affiliations:** Vascular Physiology Group, Department of Cell Biology and Physiology, University of New Mexico Health Sciences Center, Albuquerque, NM 87131, USAjoshua.sheak@cchmc.org (J.R.S.); bwalker@salud.unm.edu (B.R.W.); njernigan@salud.unm.edu (N.L.J.)

**Keywords:** pulmonary hypertension, hypoxia, mitochondria, reactive oxygen species, vasoconstriction

## Abstract

Pulmonary hypertension (PH) resulting from chronic hypoxia (CH) occurs in patients with chronic obstructive pulmonary diseases, sleep apnea, and restrictive lung diseases, as well as in residents at high altitude. Previous studies from our group and others demonstrate a detrimental role of reactive oxygen species (ROS) in the pathogenesis of CH-induced PH, although the subcellular sources of ROS are not fully understood. We hypothesized that mitochondria-derived ROS (mtROS) contribute to enhanced vasoconstrictor reactivity and PH following CH. To test the hypothesis, we exposed rats to 4 weeks of hypobaric hypoxia (P_B_ ≈ 380 mmHg), with control rats housed in ambient air (P_B_ ≈ 630 mmHg). Chronic oral administration of the mitochondria-targeted antioxidant MitoQ attenuated CH-induced decreases in pulmonary artery (PA) acceleration time, increases in right ventricular systolic pressure, right ventricular hypertrophy, and pulmonary arterial remodeling. In addition, endothelium-intact PAs from CH rats exhibited a significantly greater basal tone compared to those from control animals, as was eliminated via MitoQ. CH also augmented the basal tone in endothelium-disrupted PAs, a response associated with increased mtROS production in primary PA smooth muscle cells (PASMCs) from CH rats. However, we further uncovered an effect of NO synthase inhibition with Nω–nitro-L-arginine (L-NNA) to unmask a potent endothelial vasoconstrictor influence that accentuates mtROS-dependent vasoconstriction following CH. This basal tone augmentation in the presence of L-NNA disappeared following combined endothelin A and B receptor blockade with BQ123 and BQ788. The effects of using CH to augment vasoconstriction and PASMC mtROS production in exogenous endothelin 1 (ET-1) were similarly prevented by MitoQ. We conclude that mtROS participate in the development of CH-induced PH. Furthermore, mtROS signaling in PASMCs is centrally involved in enhanced pulmonary arterial constriction following CH, a response potentiated by endogenous ET-1.

## 1. Introduction

Chronic hypoxia (CH) occurs in patients with chronic obstructive pulmonary diseases, restrictive lung diseases, and sleep apnea, as well as in residents at high altitude. One of the detrimental outcomes of CH is pulmonary hypertension (PH, World Health Organization (WHO) Group III). Right heart failure resulting from PH leads to morbidity and mortality. Unfortunately, the current therapies for CH-associated PH are limited [1]. Therefore, a better understanding of the mechanisms leading to CH-induced PH is key to overcoming this dilemma.

Apart from pulmonary arterial remodeling [2], previous findings from our laboratory and others have identified an indispensable role of augmented vasoconstriction [2,3] in the pathogenesis of CH-induced PH [4]. Such vasoconstriction manifests as increases in both the basal resting tone and responses to endogenous vasoconstrictors resulting from increased cytosolic Ca^2+^ and Ca^2+^-sensitization mechanisms in the pulmonary arterial smooth muscle cells (PASMCs) [5,6]. This process is also facilitated by CH-induced endothelial dysfunction, which involves the increased production of vasoconstrictor and proliferative factors such as endothelin 1 (ET-1) [7,8].

Reactive oxygen species (ROS) are oxygen-derived molecules with unpaired electrons in their outer orbit. They can serve as signaling messengers during various physiological and pathological processes within the cardiovascular system [9]. Studies report that elevated ROS mediate augmented pulmonary arterial constriction [2,6,10,11], and experimental antioxidant therapies ameliorate PH in animal models [2,12,13,14,15,16]. In humans, oxidative stress is increased in high-altitude residents [17] and in pulmonary hypertensive patients [18]. In addition, a small-scale clinical trial revealed that oral antioxidant coenzyme Q improves right heart function, as assessed by echocardiography in patients with pulmonary arterial hypertension (PAH; WHO Group I) [19].

Our laboratory further characterized a pivotal role of ROS-dependent Rho kinase (ROK)-mediated myofilament Ca^2+^ sensitization in enhanced vasoreactivity in the setting of CH-induced PH [6]. Although we identified NADPH oxidase 2 (NOX2) as an enzymatic source of ROS [20], the contribution of other sources of ROS, including mitochondria, remain unclear.

Along with ATP production, mitochondria continuously generate ROS from the electron transport chain (ETC) that are physiologically important under normal conditions [21]. The redox homeostasis of mitochondria is maintained by an antioxidant system that includes superoxide dismutase 1 (SOD1) in the intermembrane space and SOD2 in the matrix. However, mitochondrial dysfunction and the resultant oxidative stress have been implicated as important contributing factors to several disease states. For example, studies support a direct role for mitochondria-derived superoxide in cardiac ischemia–reperfusion injury [22], systemic hypertension [23], and angiotensin II signaling in endothelial cells [24]. Although there is considerable debate regarding the role of mitochondrial ROS (mtROS) in acute hypoxic pulmonary vasoconstriction [25,26], there is little evidence to support a direct contribution of mtROS to CH-induced PH. Furthermore, conflicting evidence exists with respect to effects of chronic in vitro hypoxia on mtROS production in human pulmonary arterial endothelial cells [12] and murine PASMCs [27], as well as effects of targeted reduction of mtROS on indices of PH in vivo [12,27]. Interestingly, a recently published study from our laboratory revealed a critical role for mtROS signaling in enhanced vasoconstriction and PH in rat models of sleep apnea [28]. The present study therefore aimed to test the hypothesis that mtROS contribute to increased basal pulmonary arterial tone and augmented vasoconstrictor sensitivity following CH in adult rats, using cell imaging approaches and video-microscopy of pressurized small pulmonary arteries. We further assessed the significance of mtROS to the development of CH-induced PH in whole animal studies, using echocardiography, direct measurements of right ventricular pressure, and morphometric analysis of arterial remodeling.

## 2. Methods

### 2.1. Animal Model

Male Sprague-Dawley rats (200–250 g) were purchased from Envigo and allowed to acclimate for ~1 week before experimentation. Rats were divided into either control or chronic hypoxia (CH) groups. Rats designated for the CH group were housed in hypobaric chambers (P_B_ ≈ 380 mmHg; 4 weeks). Age-matched control rats were housed in the same facility under normobaric conditions (P_B_ ≈ 630 mmHg; 4 weeks; Albuquerque NM). Rats were housed 2–3/cage. The hypobaric chambers were opened 3 times/week to provide animals with clean bedding and fresh food and water. A 12:12 h light-and-dark cycle was maintained in the animal facility. All experimental protocols and surgical procedures were reviewed and approved by the Institutional Animal Care and Use Committee (IACUC) of the University of New Mexico School of Medicine (Approval Number: 19-200965-HSC).

### 2.2. Contribution of mtROS to Chronic Hypoxia-Induced Pulmonary Hypertension

#### 2.2.1. Experimental Groups

A subset of control and CH rats was administered the mitochondria-targeted antioxidant MitoQ (500 μM [29,30,31,32], MitoQ Ltd., Auckland, New Zealand) in their drinking water, beginning 1 week prior to placement of animals in the hypobaric chamber, and continuously during the 4 weeks period of CH or ambient air exposure. This protocol has previously been reported to effectively deliver MitoQ in rodent studies [29,30,31,32]. Rats designated for vehicle treatment received water only. Each cage contained 2 rats. Body weight and water consumption were measured 3 times/week. MitoQ was administered freshly dissolved in water. Animals were subjected to the following protocols.

#### 2.2.2. Echocardiography

Echocardiography (Vevo LAZR-X 3100, FUJIFILM VisualSonics, Toronto, ON, Canada) was performed prior to and following 2 and 4 weeks of normoxic or hypoxic exposure. Rats were anesthetized (2% isoflurane/98% O_2_; 1 L/min: Kent Scientific, Torrington, CT, USA) and placed on a heating table (37 °C) for electrocardiography (ECG) and assessment of respiration rate and body temperature. Cardiac output (B mode) and pulmonary artery acceleration time (pulse wave Doppler mode) were acquired using a MX250S probe in the parasternal long axis view.

#### 2.2.3. Right Ventricular and Systemic Arterial Blood Pressure Measurement

At the end of the 4 week exposure protocol, rats were anesthetized with inhaled isoflurane, as above, and placed in a supine position on a heating pad. Peak right ventricular systolic pressure (RVSP) [2] and mean arterial blood pressure (MABP) were measured using heparinized saline-filled (0.9% saline containing 10 units/mL heparin) catheters connected to an APT300 pressure transducer with a TAM-A bridge amplifier (Harvard Apparatus, Holliston, MA, USA). A curved vinyl catheter (inner diameter (i.d.) = 0.4 mm, Dural Plastics & Engineering, Auburn, Australia) was inserted through a small incision in the right jugular vein and advanced into the right ventricle to measure peak RVSP as an index of peak pulmonary arterial systolic pressure. A similar approach was used to measure MABP by inserting a polyethylene catheter (i.d.= 0.28 mm, Clay Adams, Sparks, MD, USA) in the femoral artery. All data were acquired and analyzed by a computer-based data-acquisition system (LabChart 8; AD Instruments, Colorado Springs, CO, USA). 

#### 2.2.4. Hematocrit

Hematocrit (% red blood cells) was measured in blood samples from the femoral artery collected in glass microcapillary tubes.

#### 2.2.5. Fulton’s Index

After isolation of the heart and removal of atria and great vessels, the heart was cut along the anterior and posterior interventricular groove and separated into two parts, namely right ventricle (RV) and left ventricle plus interventricular septum (LV+S). CH-induced RV hypertrophy was assessed by Fulton’s index, calculated by normalizing the weight of the RV to that of (LV+S).

#### 2.2.6. Lung Fixation and Assessment of Pulmonary Arterial Remodeling

A median sternotomy was performed in isoflurane-anesthetized rats to expose the heart and lungs. Heparin (100 units in 0.1 mL; Fresenius Kaba USA, LLC., Lake Zurich, IL, USA) was injected into the right ventricle, followed by an injection of pentobarbital sodium (20 mg/kg; Fatal Plus, Hospira, Lake Forest, IL, USA) into the left ventricle. A 13-gauge needle stub was inserted into the pulmonary artery trunk via an incision in right ventricle outflow track and secured with a silk ligature (0 USP, HOSPEQ Inc., Miami, FL, USA) placed underneath the great vessels through the pericardial transverse sinus. This pathway served as the inlet of lung perfusate, and a small hole cut into the left ventricle served as the outlet. The pulmonary vasculature was washed with 250 mL of physiological saline solution (PSS) perfusate (containing (in mM) 129.80 NaCl, 5.40 KCl, 0.83 MgSO_4_, 19.00 NaHCO_3_, 5.50 glucose, and 3.00 EGTA; all from Sigma, St. Louis, MO, USA) supplemented with 4% bovine serum albumin (BSA, wt/vol, Sigma) and 0.1 mM papaverine (Sigma), and then it was perfused with 250 mL of fixative (4% PFA with 0.1 mM papaverine). The trachea was cannulated with a 17-gauge needle stub, and the lung was inflated with fixative, at a pressure of 25 cm H_2_O. The heart/lung block was removed, immersed in 4% PFA, and stored at 4 °C until the paraffin embedding.

Paraffin-embedded lung blocks were cut into sections with a thickness of 4 µm. Following deparaffinization and rehydration, antigens were retrieved by boiling slides in buffer containing 10 mM Tris (pH 9.0), 1 mM EDTA, and 0.05% Tween20, at 90–95 °C for 15 min. Sections were placed in PBS supplemented with 2% normal goat serum and 0.1% Triton X-100 for 30 min to permeabilize the cells and block non-specific epitopes. Smooth muscle alpha-actin (α-SMA) was labeled using mouse-derived primary antibodies (1:1000, 4 °C, overnight, Sigma), followed by Alexa Fluor 488-conjugated donkey anti-mouse secondary antibody (1:800, room temperature, 2 h, Jackson ImmunoResearch, West Grove, PA, USA). Nuclei were counterstained with TO-PRO^®^-3 (0.5 µM, Thermo Fisher Scientific, Rockford, IL, USA) for 10 min at room temperature. Sections were mounted on Superfrost Plus slides (Thermo Fisher Scientific) with mounting media (10% Mowiol 4-88 (Sigma), 25% glycerol and 0.1 mL Tris; pH 8.5) and stored at 4 °C in the dark prior to imaging.

All sections were imaged using a Leica confocal microscope (TCS SP5; Wetzlar, Germany) equipped with a 63× glycerol immersion objective (numerical aperture: 1.30). Smooth muscle α-SMA detection was achieved by excitation at 488 nm, using an argon laser at a power of 20%, and emissions were filtered through an Acousto-Optical Beam Splitter (AOBS; Leica) crystal to collect wavelengths of 500–550 nm. Nuclear staining with TO-PRO^®^-3 was detected with a HeNe laser (633 nm) at a power of 5%, and emissions were passed through an AOBS (Leica) to collect a bandwidth of 650–750 nm. The scanner was set to a pinhole of 76.8 µm, a resolution of 8 bits, and a speed of 400 Hz. All images were obtained using a 1024 × 1024 frame and frame averaging of 3 scans.

Images were analyzed by ImageJ software Version 1.51 (NIH). Arterial wall muscularization was determined in small pulmonary arteries. A threshold of 30 to 255 gray levels (maximum for 8-bit images) was applied to all Images. For each vessel, a region of interest (ROI) was drawn along the exterior of the threshold smooth muscle α-SMA labeled area in fully muscularized pulmonary arteries. Percent muscularization was expressed as a percentage of the α-SMA-labeled area to total ROI area [2]. Vessel diameter was calculated from the circumference of the ROI. Percent muscularization of 20–30 randomly selected small pulmonary arteries (vessel diameter less than 120 μm with average diameter of ~50 μm) was averaged to represent a single “*n*” (animal) for subsequent statistical analysis.

### 2.3. Pressure Myography

Rats were euthanized with pentobarbital sodium (200 mg/kg, i.p.), and the left lung was removed via midline thoracotomy. Small pulmonary arteries (PAs, i.d.= ~100–200 μm) were isolated, cannulated on glass microcannula, and secured in a vessel perfusion chamber (Living Systems Instrumentation, Burlington, VT, USA). Isolated pulmonary arteries were pressurized to 12 mmHg, using a servo-controlled peristaltic pump (Living Systems Instrumentation). Vessel inner diameter was measured via videomicroscopy (Nikon Eclipse TS100 microscope; Tokyo, Japan). Experiments were conducted in endothelium-intact pulmonary arteries in the presence or absence of the NO synthase inhibitor Nω–nitro-L-arginine (L-NNA, 300 µM [33], Sigma), or in endothelium-disrupted PAs, achieved by passing a fiber of moose main through the lumen of the vessel [2].

Pressurized pulmonary arteries were continuously superfused (5 mL/min) with PSS (37 °C; containing the following (in mM): 129.80 NaCl, 5.40 KCl, 0.83 MgSO_4_, 19.00 NaHCO_3_, 1.80 CaCl_2_, and 5.50 glucose; all from Sigma). The superfusate was bubbled with a gas mixture containing 6% CO_2_/10% O_2_/balance N_2_ to approximate the normoxic PO_2_ to which these arteries are exposed in vivo. Previous studies from our laboratory indicate that this gas mixture yields an approximate superfusate with pH = 7.40, PO_2_ = 57 mmHg, and PCO_2_ = 31 mmHg [33]. Following a 20 min equilibration period, either basal tone or vasoconstriction to exogenous ET-1was assessed.

#### 2.3.1. Basal Tone Measurement in Small Pulmonary Arteries

The contribution of mtROS to basal tone was measured in both endothelium-intact and endothelium-disrupted PAs. For endothelium-intact PAs, L-NNA was added in the superfusate after the 20 min equilibration period. Baseline i.d. was recorded both prior to and after the administration of L-NNA when the i.d. had stabilized. For endothelium-disrupted PAs, baseline i.d. was recorded after the 20 min equilibration period.

After baseline i.d. was recorded, vessels were superfused with Ca^2+^-free PSS (containing (in mM) 129.80 NaCl, 5.40 KCl, 0.83 MgSO_4_, 19.00 NaHCO_3_, 5.50 glucose, and 3.00 EGTA; all from Sigma) for 30 min, followed by the administration of the Ca^2+^ ionophore ionomycin (3 μM, Sigma) to achieve maximal i.d. of pulmonary arteries. Basal tone is defined as the percentage of the maximally dilated (Ca^2+^-free) inner diameter, i.e., basal tone = [(Ca^2+^-free i.d. − Baseline i.d.)/Ca^2+^-Free i.d.] × 100%. The contribution of mtROS to basal tone was determined via the administration of MitoQ (1 μM [34], MitoQ Ltd.), the mitochondrial superoxide scavenger MitoTEMPO (200 μM [35], Sigma), or the vehicle.

#### 2.3.2. Vasoconstrictor Response to ET-1 in Small Endothelium-Disrupted PAs

ET-1-induced vasoconstriction was detected in PAs with endothelium disruption to exclude the influence of endogenous ET-1. Baseline i.d. was recorded after 20 min equilibration. Then, a series doses of ET-1 (10^−11^ to 10^−7^ M, Sigma) were added to the superfusate. Reduction in i.d. in response to each dose was calculated 10 min after adding ET-1 or until the i.d. was stable. Vasoconstriction was expressed as the percentage of baseline i.d. reduction in response to ET-1. Experiments were performed in the presence and absence of MitoQ (1 μM, MitoQ Ltd.) to study the role of mtROS in this response.

### 2.4. MtROS Detection in Pulmonary Arterial Smooth Muscle Cells (PASMCs)

#### 2.4.1. Establishment of Primary PASMC Cultures

Rats were euthanized with pentobarbital sodium (200 mg/kg, i.p.), and the lungs were collected. Intrapulmonary arteries (2nd-to-5th order) were dissected from both the left and right lungs and placed in Hank’s balance salt solution (HBSS, Gibco, Rockford, IL, USA) supplemented with 20 μM Ca^2+^ and 1% (*v*/*v*) pen/strep (Gibco). Harvested PAs were digested using an enzyme mixture containing papain (2 mg/mL, Sigma), type I collagenase (6 mg/mL, Sigma), DL-dithiothreitol (1 mg/mL, Sigma), and BSA (2 mg/mL, Sigma) at 37 °C for 30 min [2]. Enzymatically digested PAs were transferred to a 70 µm cell strainer and washed with 5 mL Ca^2+^-free HBSS. The vessels were then added to a 1.5 mL sterile Eppendorf tube containing 1 mL of Ca^2+^-free HBSS and triturated using a sterile pipette tip. The cells were grown in Ham’s F-12 media (Gibco) with 5% (*v*/*v*) fetal bovine serum (FBS, Gibco) and 1% (*v*/*v*) pen/strep (Gibco) for 3–4 days in the incubator (Panasonic; Osaka, Japan) operated at 37 °C and 5% CO_2_.

#### 2.4.2. Basal mtROS Measurement in PASMCs

PASMCs harvested from control and CH rats were grown on 12 mm coverslips (VWR) and placed in 24-well plates (Corning, Kennebunk, ME, USA ). Cells were cultured for 3-4 days, as described above. PASMCs were pretreated with MitoQ (1 μM, MitoQ Ltd.), MitoTEMPO (200 μM), or the vehicle for 1 h before the detection of mtROS. MtROS levels were determined by incubating PASMCs with the fluorescent mitochondrial superoxide indicator MitoSOX [36] (5 μM [37], Invitrogen, Rockford, IL, USA), as well as mitochondrial marker MitoTracker (1 μM, Invitrogen), for 20 min in the incubator. After rinsing with PBS (Gibco), cells were fixed with 4% paraformaldehyde for 15 min at room temperature in the dark. Fixed cells were mounted with ProLong Gold Antifade reagent (Invitrogen) and dried in the dark, overnight, at 4 °C. Slides were examined the following day, and images were taken in 6-to-10 randomly selected views per coverslip.

#### 2.4.3. ET-1 Induced mtROS Production in PASMCs by Live Cell Imaging

PASMCs were cultured in glass-bottom microwell dishes (35 mm Petri dish with 14 mm microwell, MatTek Corporation, Ashland, MA, USA) for 2 days, using Ham’s F-12 media (Gibco) with 5% (*v*/*v*) fetal bovine serum (FBS, Gibco) and 1% (*v*/*v*) pen/strep (Gibco). Cells were serum-starved (0.3% FBS) overnight before experimentation. To study the regulatory role of ET-1 in mtROS production, live PASMCs were loaded with MitoSOX (10 µM, Invitrogen) and MitoTracker (1 µM, Invitrogen) for 20 min in HEPES buffer (containing the following (in mM): 130.00 NaCl, 4.00 KCl, 1.20 MgSO_4_, 4.00 NaHCO_3_, 10.00 HEPES, 1.18 KH_2_PO_4_, 1.80 CaCl_2_, and 6.00 glucose; pH adjusted to 7.40 with NaOH; all from Sigma). After a baseline image was taken, the vehicle or ET-1 (1 nM, Sigma) was added to the dish, and a series images were taken from the same field of view every 10 min for a 30 min period. Cells were maintained at 37 °C in a humidified environment throughout the loading and imaging process.

#### 2.4.4. Confocal Microscopy and Image Quantification

Images were collected using a 63× glycerol immersion objective (numerical aperture: 1.30) on a Leica confocal microscope (TCS SP5). MitoSOX was excited using an argon laser (514 nm) at a power level of 20%, and emissions were filtered through an AOBS (Leica) to collect wavelengths of 525–600 nm. The MitoTracker was excited using a HeNe laser (633 nm) at 5% power, and emissions were processed through an AOBS (Leica) to collect bandwidths of 650–750 nm. The scanner was set to a pinhole diameter of 102.9 µm, with a resolution of 8 bits and speed of 400 Hz. All images were obtained using a 2048 × 2048 frame and frame averaging of 3 scans.

The mean fluorescence intensity (MFI) of MitoSOX in regions of interest (ROIs) was assessed using ImageJ software Version 1.51 (NIH). After mitochondria within a cell were identified in the MitoTracker channel, a rectangular ROI was drawn at the MitoTracker positive location in the MitoSOX channel. For basal mtROS measurements, the MitoSOX fluorescence intensities in a total of 50–70 cells from the same animal were quantified and averaged for statistical analysis. For ET-1-induced mtROS production, a single view was imaged before (baseline) and every 10 min during the 30 min ET-1 or vehicle incubation period. The MitoSOX fluorescence intensities of the same 6 randomly selected cells were quantified and averaged for each image. Changes in MitoSOX fluorescence intensity were represented by normalizing MFI at each time point to baseline MFI.

### 2.5. MtROS in Acutely Isolated Endothelial Sheets

Pulmonary arteries (3rd–5th order, 100-to-400 μm i.d.) were isolated from the left lung in HEPES buffer. PAs were cut open longitudinally to expose the endothelium and then digested in HEPES buffer supplemented with DL-dithiothreitol (0.2 mg/mL [38], Sigma), papain (0.2 mg/mL [38], Sigma), and BSA (2 mg/mL [38], Sigma) for 45 min at 37 °C. Vessels were rinsed in buffer and cut into small pieces under a dissection microscope (Nikon; Tokyo, Japan). Vessel segments were transferred to a new Eppendorf tube containing 0.5 mL HEPES buffer supplemented with 2 mg/mL BSA (Sigma). Endothelial sheets were released by gently pipetting the digested vessels up and down 10–15 times with a 1000 µL pipette tip. A few drops of solution containing endothelial sheets were placed on poly-L-lysine-coated slides (Electron Microscopy Sciences, Hatfield, PA, USA) and allowed to attach for 30 min in the cell incubator operated at 37 °C and 5% CO_2_. Sheets were treated with the vehicle, L-NNA (300 μM), or MitoQ (1 μM) for 30 min in the incubator. MtROS levels were detected by incubating endothelial sheets with MitoSOX (5 μM, Invitrogen) for 20 min in the incubator. After fixation with 4% PFA for 15 min at room temperature, slides were mounted with ProLong Gold Antifade reagent (Invitrogen) and dried overnight at 4 °C.

Images were collected using a Leica confocal microscope (TCS SP5) equipped with a 63× glycerol immersion objective. The confocal settings were identical to those described above. At least 3 different endothelial sheets were imaged for each treatment. The mean fluorescence intensity (MFI) of MitoSOX was quantified by ImageJ software Version 1.51 (NIH) after applying a threshold of 30-to-255 gray levels (maximum for 8-bit images) to the image. All endothelial sheets imaged for a specific treatment were averaged to enter statistical analysis.

For endothelial cell verification, sheets were first fixed with 4% PFA for 15 min at room temperature. Nuclei were labeled with SYTOX Green (1 μM, Invitrogen) for 20 min at room temperature in the dark, followed by Lycopersicon Esculentum Lectin, DyLight 649 staining (20 µg/mL, Vector Laboratories, Newark, CA, USA) for 30 min at room temperature in the dark. Slides were mounted with ProLong Gold Antifade reagent (Invitrogen) and dried overnight at 4 °C. Images were taken with a Leica confocal microscope (TCS SP5) equipped with a 63× glycerol immersion objective. SYTOX Green was excited by an argon laser (488 nm) at a power level of 20%, and emissions were filtered through an AOBS (Leica) to collect wavelengths of 500–550 nm. DyLight 649 was excited with a HeNe laser (633 nm) at 15% intensity, and emissions were processed through an AOBS (Leica) to collect bandwidths of 650–750 nm. The scanner was set to a pinhole diameter of 102.9 µm, a resolution of 8 bits, and a scan speed of 400 Hz. All images were obtained using a 2048 × 2048 frame and frame averaging of 3 scans.

### 2.6. Statistical Analysis

Values of “*n*” refer to the numbers of animals utilized for each experiment. Data are expressed as means ± standard error of the mean (SEM). Data expressed as percentages were arcsine-transformed before statistical tests to allow for a parametric analysis. Data were analyzed using Student’s *t*-test, one-way analysis of variance (ANOVA), or two-way ANOVA. If differences were detected via ANOVA, individual groups were compared using Tukey’s post hoc test. A *p* < 0.05 was considered statistically significant. All statistical analyses were conducted using SigmaPlot 14.5 software. This study did not utilize data exclusion criteria, and such criteria were not set a priori.

## 3. Results

### 3.1. Involvement of MtROS in CH-Induced Pulmonary Hypertension

**Figure 1:**
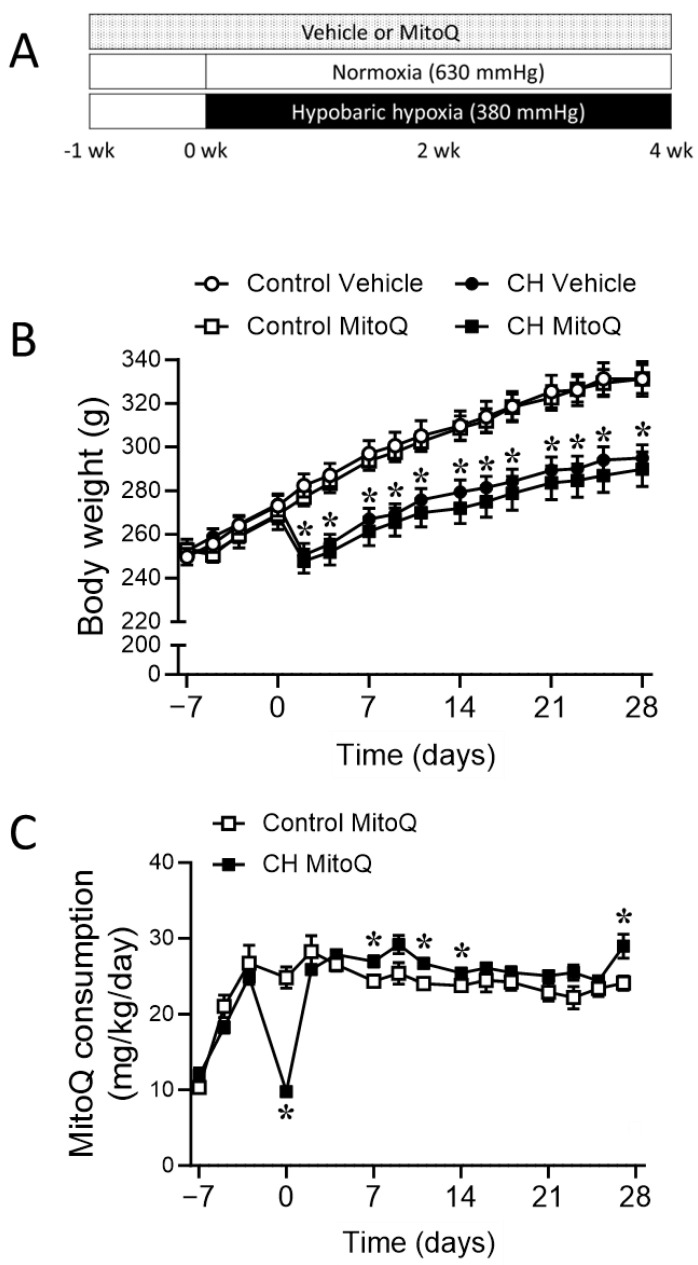
Chronic MitoQ administration. (**A**) Schematic protocol. MitoQ (500 μM) or vehicle was delivered via drinking water beginning 1 week prior to placement of animals in the hypobaric chamber and continuously during the 4-week CH or ambient air exposure period. (**B**) Body weight and (**C**) MitoQ consumption were monitored throughout the study. *n* = 7–8 animals/group; * *p* < 0.05 vs. respective control; analyzed using two-way ANOVA, followed by Tukey’s post hoc test (panel **B**) or unpaired *t*-test (panel **C**).

To determine the significance of mtROS in the development of CH-induced PH, we delivered the mitochondria-targeted antioxidant MitoQ (500 µM in drinking water [29,30,31,32]) to rats beginning 1 week prior to placement in the hypobaric chamber and continuously during the 4-week CH or ambient air exposure (Figure 1A). Consistent with our previous observations [2], the rats exhibited significant decreases in body weight (Figure 1B) and water/MitoQ intake (Figure 1C) during the first several days in the hypobaric chamber. Despite a similar rate of weight gain after day 3, rats exposed to hypoxia maintained significantly lower body weights compared to normoxic controls. The administration of MitoQ did not alter body weight over the course of study (Figure 1B) in either group. Rats housed in the hypobaric chamber consumed slightly more MitoQ compared to normoxic controls after recovery from an initial water consumption drop during the first two days of hypoxic exposure (Figure 1C).

**Figure 2:**
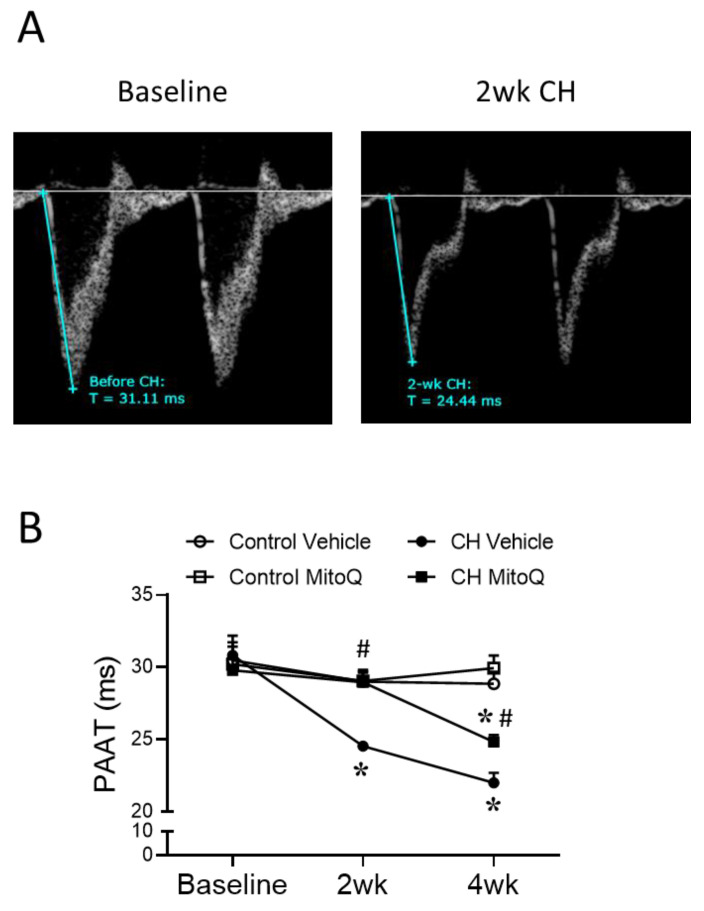
MitoQ limits the effect of CH to reduce PAAT. Pulmonary artery acceleration time (PAAT) was monitored by echocardiography as an estimation of pulmonary arterial pressure (inverse relationship). Animals were studied under anesthesia by 2% isoflurane inhalation. Measurements were taken before and after 2 or 4 weeks of normoxic or hypoxic exposure. (**A**) Representative traces of PAAT. (**B**) PAAT in rats treated with vehicle or MitoQ (500 µM) in drinking water. n = 5–8 animals/group; * *p* < 0.05 vs. respective control, and # *p* < 0.05 vs. CH vehicle; analyzed using two-way ANOVA, followed by Tukey’s post hoc test.

Echocardiography was conducted biweekly to monitor the pulmonary artery acceleration time (PAAT), which is a reliable estimation of pulmonary artery pressure [39], along with cardiac output. Decreases in PAAT and a characteristic PH waveform were observed at both 2 and 4-week time points of CH exposure in vehicle-treated rats (Figure 2A), indicative of elevated pulmonary arterial pressure. The CH-induced decrease in PAAT was prevented by MitoQ treatment after 2 weeks of hypoxic exposure, and it was significantly attenuated after 4 weeks (Figure 2B). Neither cardiac output nor heart rate (Table 1) was altered by the CH exposure or MitoQ intake.

**Figure 3:**
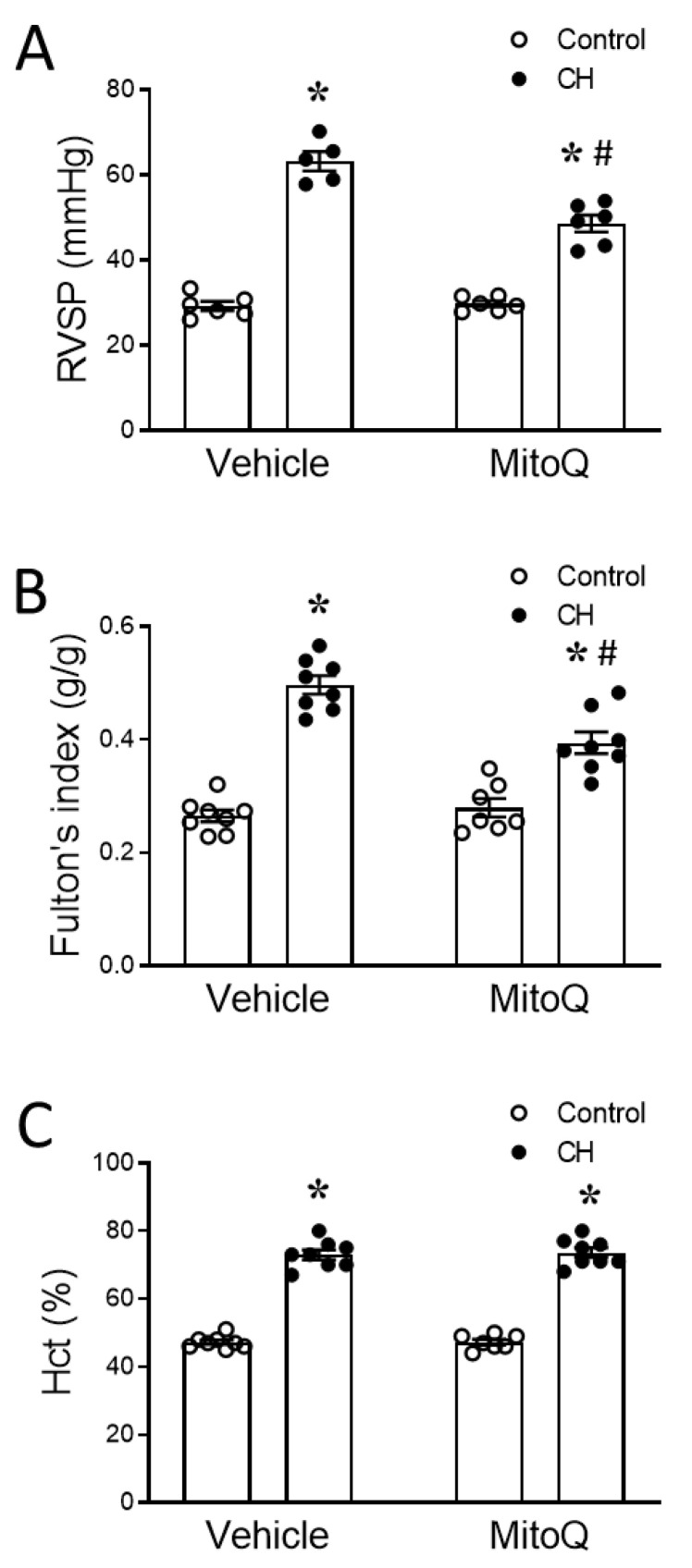
MitoQ attenuates CH-induced increases in RVSP and right ventricular hypertrophy. (**A**) Peak RVSP, (**B**) Fulton’s index (right ventricle wt. normalized to left ventricle plus septum wt. (RV/LV+S)), and (**C**) hematocrit (Hct) in control and CH rats treated with vehicle or MitoQ. RVSP was measured with an indwelling catheter in isoflurane-anesthetized rats. *n* = 5–8 animals/group; * *p* < 0.05 vs. respective control, and # *p* < 0.05 vs. CH vehicle; analyzed using two-way ANOVA, followed by Tukey’s post hoc test.

At the conclusion of the 4-week exposure period, the peak right ventricular systolic pressure (RVSP), right ventricular (RV) hypertrophy, and pulmonary arterial remodeling were measured in each group to further evaluate the contribution of mtROS to CH-induced PH. Consistent with the echocardiography findings, CH-induced increases in peak RVSP (Figure 3A) and right ventricular hypertrophy (Figure 3B) were both attenuated by MitoQ. In contrast, the polycythemic response to CH was not altered by MitoQ treatment (Figure 3C). MitoQ was without effect on these variables in the control rats.

The mean systemic arterial pressure (MSAP) and heart rate (HR) were unaltered by CH exposure (Table 2). In addition, MitoQ treatment had no effect on MSAP or HR in both control and CH animals.

**Figure 4:**
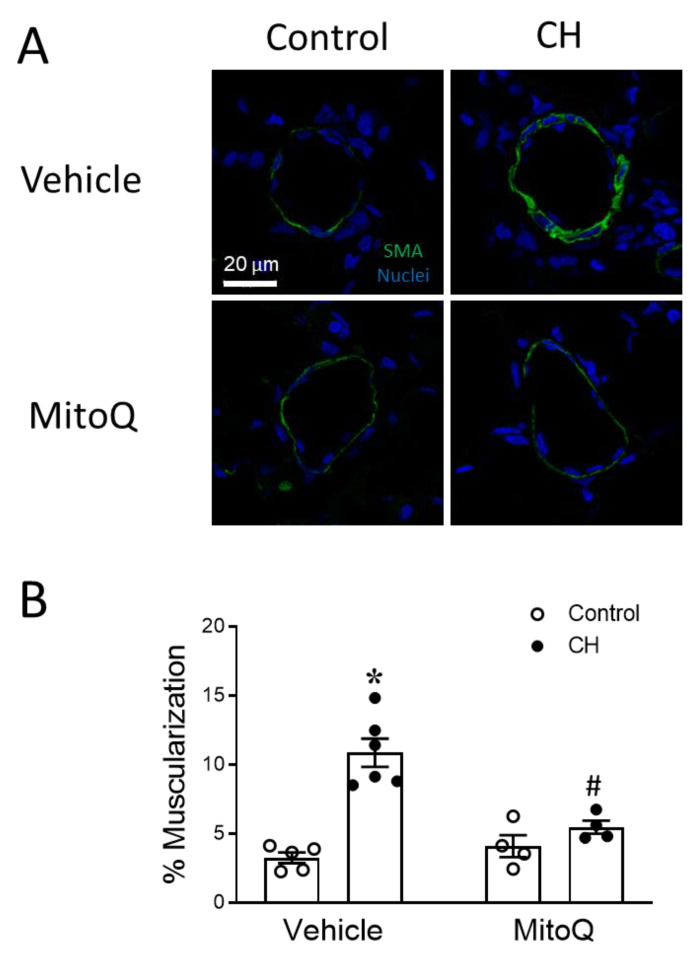
MtROS mediate the pulmonary arterial remodeling response to CH. (**A**) Representative immunofluorescence images of small pulmonary arteries (green = smooth muscle α-actin; blue = TOPRO3) and (**B**) % muscularization of arteries from control and CH rats administered vehicle or MitoQ (mean diameter of ~50 μm from 20–30 vessels/animal/group). *n* = 4–6 animals/group; * *p* < 0.05 vs. control vehicle, and # *p* < 0.05 vs. CH vehicle; analyzed using two-way ANOVA, followed by Tukey’s post hoc test.

The arterial wall muscularization in small pulmonary arteries (PAs) was assessed as an index of arterial remodeling. The percent muscularization was calculated by normalizing the area of smooth muscle α-SMA labeling to total vessel area. CH exposure increased the muscularization of PAs, which was prevented by MitoQ (Figure 4). Collectively, these results support a contribution of mtROS to the pathogenesis of CH-induced PH.

### 3.2. MtROS Mediate CH-Induced Increases in Pulmonary Arterial Tone

**Figure 5:**
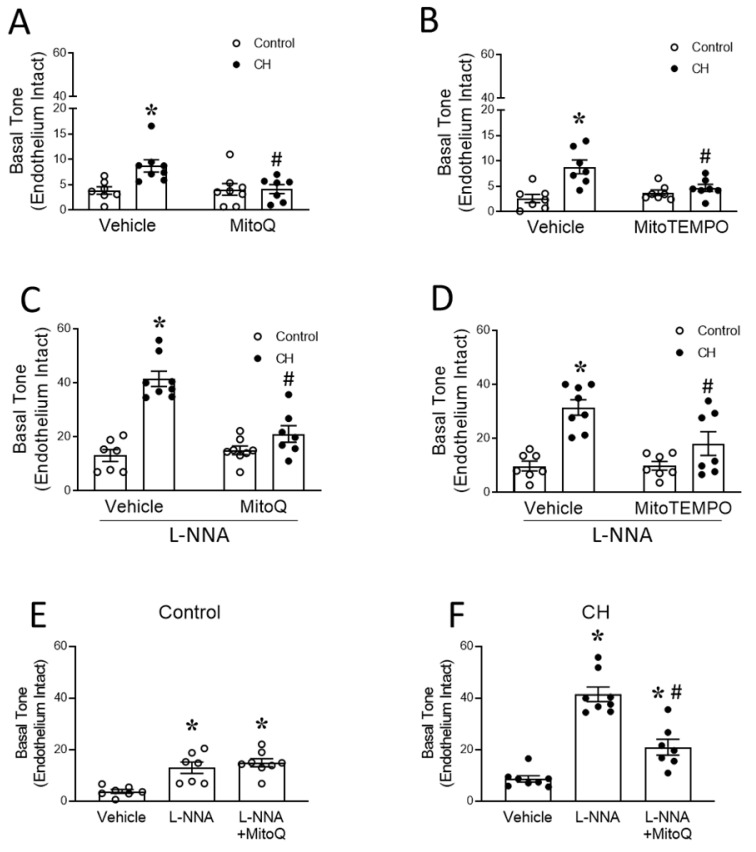
MtROS are responsible for CH-induced increases in pulmonary arterial tone. Basal tone (% passive (Ca^2+^-free) i.d.) in isolated small pulmonary arteries with intact endothelium. Experiments were performed in the absence (**A**,**B**) and presence of the NO synthase inhibitor L-NNA (300 μM, **C**,**D**). The contribution of mtROS to basal tone was studied by utilizing the mitochondria-targeted antioxidants MitoQ (1 μM, panels **A**,**C**) or MitoTEMPO (200 μM, panels **B**,**D**). *n* = 7–8 animals/group; * *p* < 0.05 vs. control vehicle, and # *p* < 0.05 vs. CH vehicle; analyzed using two-way ANOVA, followed by Tukey’s post hoc test. Panels (**E**,**F**) depict the reorganization of data from panels (**A**–**C**), showing that basal tone was augmented by acute NOS inhibition, a response dependent on mtROS in arteries from CH rats but not control arteries. *n* = 7–8 animals/group; * *p* < 0.05 vs. vehicle, and # *p* < 0.05 vs. L-NNA; analyzed using one-way ANOVA, followed by Tukey’s post hoc test.

Given the importance of the basal pulmonary vascular tone to CH-induced PH [4], we next evaluated the role of mtROS in mediating enhanced basal PA tone following CH, using pressure myography of endothelium-intact small PAs (~150 µm inner diameter). The arteries from CH rats displayed a greater basal tone compared to those of the controls, and this response to CH was prevented by either MitoQ (Figure 5A) or the mitochondrial-targeted superoxide scavenger MitoTEMPO (Figure 5B). In contrast, MitoQ did not alter the tone in the control arteries.

The inhibition of endothelial NO synthase (eNOS) with L-NNA largely increased the basal tone in both groups (Figure 5C–F). However, both MitoQ and MitoTEMPO prevented this effect of L-NNA only in the arteries of CH rats. Additional experiments in CH arteries revealed that MitoQ significantly lowered the basal tone compared to TPP (negative control for MitoQ and MitoTEMPO), both in the presence (TPP: 37.4 ± 1.8%; MitoQ: 16.1 ± 3.0% passive i.d.; *n* = 4 rats/group; *p* < 0.05) and absence (TPP: 8.0 ± 1.9%; MitoQ: 2.8 ± 0.3% passive i.d.; *n* = 4 rats/group; *p* < 0.05) of L-NNA. Furthermore, the basal tone in the presence of TPP was similar to that observed in vehicle-treated arteries, suggesting that MitoQ and MitoTEMPO prevent CH-induced vasoconstriction through their actions as mitochondrial antioxidants rather than through the nonspecific effects of their polar head group, TPP. Collectively, these findings demonstrate that the inhibition of eNOS accentuates the mtROS-dependent vasoconstriction in PAs from CH rats, an effect that is absent in control animals.

### 3.3. PASMC-Derived mtROS Contribute to Elevated PA Tone following CH

**Figure 6:**
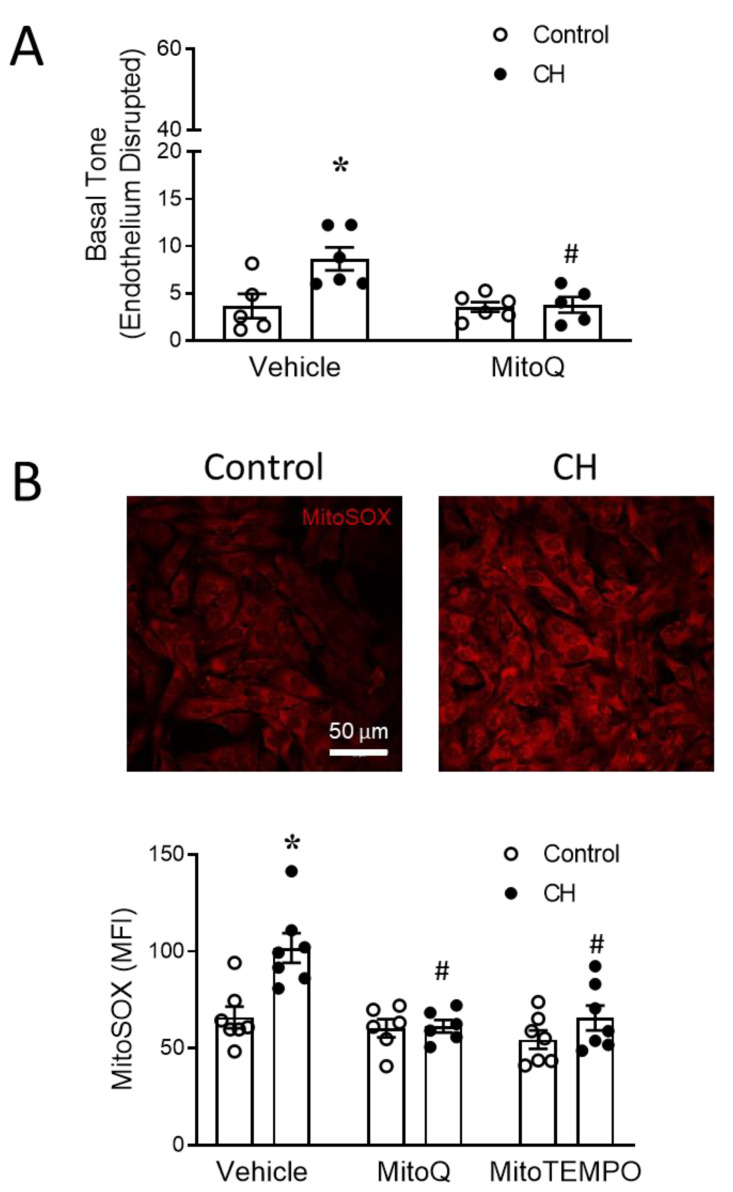
CH increases PASMC mtROS generation leading to greater basal pulmonary arterial tone. (**A**) Basal tone in endothelium-disrupted small pulmonary arteries from control and CH rats in the presence of MitoQ (1 μM) or vehicle. n = 5–6 animals/group. (**B**) Representative images of MitoSOX (5 μM) fluorescence in primary cultures of PASMCs from control and CH rats (**top**), and MitoSOX mean fluorescence intensity (MFI) (**bottom**) from cells treated with the mitochondria-targeted antioxidant MitoQ (1 μM) or the mitochondrial superoxide scavenger MitoTEMPO (200 μM). *n* = 6–7 animals/group; * *p* < 0.05 vs. control vehicle, and # *p* < 0.05 vs. CH vehicle; analyzed using two-way ANOVA, followed by Tukey’s post hoc test.

The smooth muscle and endothelium each represent potential sources of mtROS mediating increased basal arterial constriction following CH. To distinguish between the two, we first repeated the protocols described above in endothelium-intact vessels, but now using vessels in which the endothelium was disrupted by passing a fiber of moose mane through the lumen as previously described [6]. Similar to endothelium-intact arteries (Figure 5), the endothelium-disrupted PAs from CH rats exhibited a greater resting tone in comparison to those from normoxic controls, as previously reported [20]. Furthermore, this response was abolished by MitoQ (Figure 6A).

The determination of PASMCs as a source of mtROS was evaluated in short-term (3–4 days) primary cultures of PASMCs from control and CH rats, using the fluorescent mitochondrial superoxide indicator MitoSOX. MitoSOX fluorescence intensity was significantly higher in PASMCs from CH rats versus those from the controls, a response that was prevented by both MitoQ and MitoTEMPO (Figure 6B).

Together, these results support the role of CH in increasing mtROS production in PASMCs, leading to an enhanced basal tone. However, it is noteworthy that the constriction of arteries collected from CH rats in the presence of L-NNA (41.4 ± 2.8% passive i.d.; *n* = 8 rats) was markedly greater (*p* < 0.05) than that observed following endothelial disruption (8.6 ± 1.2% passive i.d.; *n* = 6 rats). This difference does not appear to be a consequence of potentially impaired vessel viability resulting from the endothelial disruption protocol, since the control experiments revealed that endothelial disruption significantly augments UTP (5 µM)-induced constriction compared to endothelium-intact arteries (intact 33.4 ± 3.5% baseline i.d.; disrupted 44.7 ± 3.5% baseline i.d.; *n* = 5 rats/group; *p* < 0.05), a response expected due to the loss of the basal vasodilatory influence of endothelium-derived NO. As such, these findings indicate a previously undescribed role for L-NNA to unmask a potent endothelial vasoconstrictor influence that mediates mtROS-dependent vasoconstriction following CH.

### 3.4. Endogenous ET-1 Contributes to Enhanced MtROS-Dependent Basal Tone following CH

**Figure 7:**
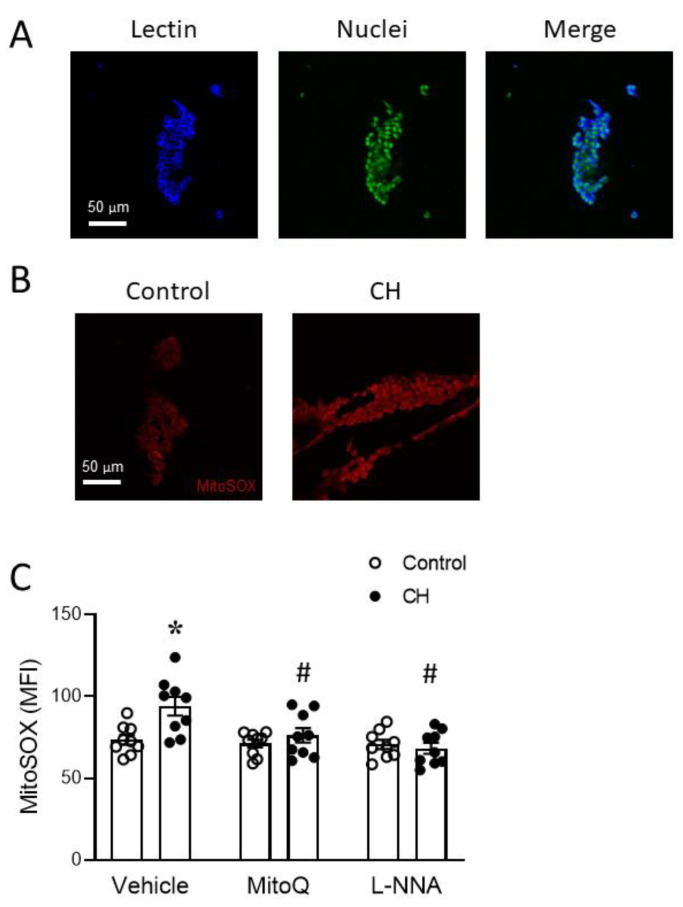
CH increases MtROS levels in acutely isolated pulmonary artery endothelial cell sheets. (**A**) Sheets were positive for the endothelial marker lectin (in blue). Nuclei were labeled with SYTOX Green. (**B**) Representative images of MitoSOX fluorescence and (**C**) MitoSOX mean fluorescence intensity (MFI) in pulmonary artery endothelial cell sheets isolated from intrapulmonary arteries (3rd–5th order, 100-to-400 μm i.d.) of control and CH rats. Experiments were conducted in the presence and absence of MitoQ (1 μM) or L-NNA (300 μM). *n* = 9 animals/group; * *p* < 0.05 vs. control vehicle, and # *p* < 0.05 vs. CH vehicle; analyzed using two-way ANOVA, followed by Tukey’s post hoc test.

The ability of the endothelium to exert a mtROS-dependent vasoconstrictor influence following CH could be explained by enhanced endothelial mtROS production or, rather, the ability of the endothelium to potentiate mtROS production by the smooth muscle. To address these possibilities, we next measured mtROS production using MitoSOX in endothelial sheets (Figure 7A) acutely isolated from both control and CH rats. Similar to the ability of CH to increase PASMC mtROS production (Figure 6B), we observed greater MitoSOX fluorescence in endothelial cells from CH rats compared to those of the control animals (Figure 7B,C). Furthermore, MitoQ prevented this response to CH, while having no significant effect in control cells. However, eNOS inhibition by L-NNA similarly abolished the effect of CH to increase mtROS (Figure 7C).

Together, our findings that L-NNA greatly enhances mtROS-dependent basal tone following CH (Figure 5) while simultaneously lowering endothelial mtROS levels suggest that endothelial mtROS are an unlikely candidate to explain the endothelial potentiation of mtROS-dependent vasoconstriction observed in CH arteries.

**Figure 8:**
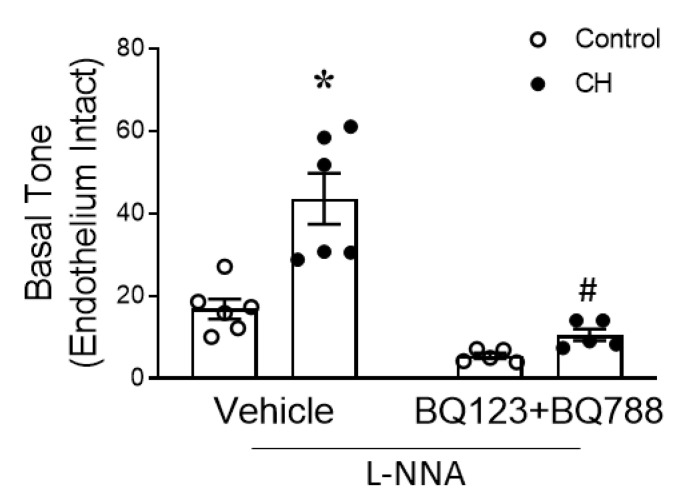
Endogenous ET-1 contributes to enhanced mtROS-dependent basal tone following CH. Basal tone in endothelium-intact small pulmonary arteries in the combined presence or absence of ET_A_ and ET_B_ receptor antagonists (BQ123 and BQ788, respectively; 10 μM each). Experiments were performed in the presence of L-NNA (300 μM). *n* = 5–6 animals/group; * *p* < 0.05 vs. control vehicle, and # *p* < 0.05 vs. CH vehicle; analyzed using two-way ANOVA, followed by Tukey’s post hoc test.

Based on these observations, we next explored an alternative possibility that the endothelium mediates a vasoconstrictor influence following CH that acts on the underlying smooth muscle to enhance mtROS-dependent vasoconstriction. Considering the well-recognized role of the endogenous vasoconstrictor and mitogen ET-1 in PH [7,8,40,41,42], we next hypothesized that pulmonary artery endothelial cell (PAEC)-derived ET-1 potentiates mtROS-dependent increases in basal tone following CH. This hypothesis was tested by examining the effects of the ET_A_ and ET_B_ receptor blockers, BQ123 and BQ788, on basal tone in endothelium-intact PAs from each group. Consistent with our hypothesis, we found that the combined administration of BQ123 and BQ788 abolished the increase effect that CH has on the basal tone (Figure 8).

**Figure 9:**
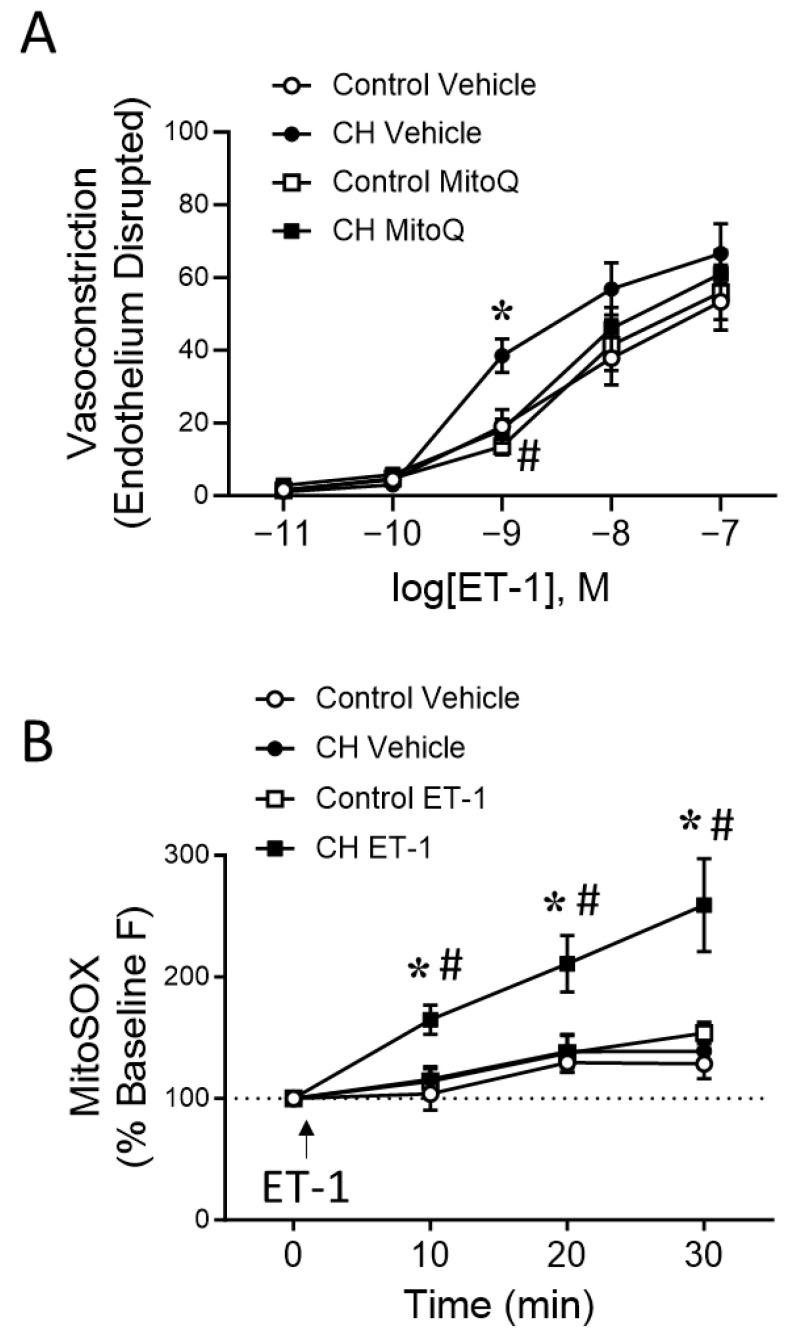
ET-1 stimulates mtROS-dependent vasoconstriction in endothelium-disrupted pulmonary arteries and mtROS production in PASMCs from CH rats. (**A**) Vasoconstrictor responses (% baseline i.d.) to ET-1 (10^−11^–10^−7^ M) in endothelium-disrupted, pressurized pulmonary arteries from control and CH rats in the presence of MitoQ (1 μM) or vehicle. *n* = 5–6 animals/group; * *p* < 0.05 vs. control vehicle, and # *p* < 0.05 vs. CH vehicle; analyzed via two-way ANOVA, followed by Tukey’s post hoc test, at each concentration. (**B**) Time-dependent changes in MitoSOX (10 μM) fluorescence (% change from baseline) in response to ET-1 (10^−9^ M) or vehicle (administered at time zero) in primary cultures of PASMCs from control and CH rats. *n* = 6 animals/group. * *p* < 0.05 vs. control ET-1, # *p* < 0.05 vs. CH vehicle; analyzed by two-way ANOVA followed by Tukey’s post hoc test at each time point.

The direct role of ET-1 in mediating mtROS-dependent PA constriction was further evaluated by determining the effects of MitoQ on vasoconstrictor responses to exogenous ET-1 (10^−11^ to 10^−7^ M) in endothelium-disrupted PAs. CH exposure augmented vasoconstriction to 10^−9^ M ET-1, which was prevented by MitoQ (Figure 9A). In contrast, vasoconstrictor responses to ET-1 in control arteries were unaffected by MitoQ. In agreement with these findings, ET-1 (10^−9^ M) significantly increased mtROS production in transiently cultured PASMCs from CH rats, while having no effect on the controls (Figure 9B).

These results collectively indicate that endogenous ET-1 promotes PA constriction through mtROS signaling in PASMCs following CH.

## 4. Discussion

The overall objective of this study was to evaluate the role of mtROS in spontaneous pulmonary arterial tone and enhanced vasoconstrictor reactivity following CH. Furthermore, we assessed the contribution of mtROS to the development of PH in a clinically relevant rat model of hypoxic PH. The major findings of this study are as follows: (1) mtROS contribute to the progression of CH-induced PH, right ventricular hypertrophy, and pulmonary arterial remodeling; (2) CH increases mtROS levels in PASMCs independent of endothelial influences, leading to enhanced basal arterial tone; (3) the endothelium markedly potentiates mtROS-dependent increases in arterial tone following CH; and (4) this effect of the endothelium is mediated by ET-1-induced stimulation of PASMC mtROS production. Collectively, these findings strongly support the overall concept that mtROS participate in both the arterial remodeling and vasoconstrictor components of CH-induced PH. Furthermore, this vasoconstrictor response to CH is largely dependent on mtROS signaling in PASMCs and is potentiated by endogenous ET-1.

Previous work from our laboratory has extensively investigated the contribution of ROS to the vasoconstrictor and arterial remodeling components of CH-induced PH [2,20,43]. Such studies have employed DHE to document the effects of CH in regard to increasing ROS production in intact pulmonary arteries and primary cultures of pulmonary vascular smooth muscle cells [2,20,43]. Prominent sources of ROS in the vasculature include NOX isoforms, mitochondria, xanthine oxidase, uncoupled eNOS, cytochrome P450, and cyclooxygenase. Of these, NOX isoforms have been the major sources of ROS implicated in the development of PH. Liu and colleagues [44] discovered that NOX2-derived ROS are increased after CH, and the knockout of NOX2 prevents CH-induced PH. NOX2-derived ROS are also critically involved in enhanced pulmonary arterial vasoconstrictor reactivity following CH, despite unaltered NOX2 protein expression [43]. However, controversy exists regarding the role of NOX4 in CH-induced PH [45,46]. For example, using female mice only and a hypoxia exposure protocol of 10% O_2_ for 15 days, Hood et al. [46] showed that RVSP is diminished when NOX4 is genetically deleted. In contrast, utilizing a 21-day 10% O_2_ hypoxia protocol, Veith et al. [45] reported that both global and inducible NOX4 knockout mice exhibit similar elevations in RVSP as WT mice. The reason for these discrepant results is not clear, but it may reflect the different animal models used. Interestingly, Rudyk et al. [47] reported that oxidation of protein kinase G Iα, potentially by NOX4-derived H_2_O_2_, is an endogenous protective mechanism in the development of PH during CH.

While mtROS have a well-established role in acute oxygen sensing and hypoxic vasoconstriction [48,49], their contribution to pulmonary hypertension remains enigmatic. Interestingly, recent studies by Suresh and colleagues demonstrated a novel role of mtROS in mediating mitochondrial fission, decreasing mitochondrial respiration, and proliferating pulmonary microvascular endothelial cells in the Sugen-hypoxia model of PAH [50]. However, previous studies addressing the role of mtROS in CH-induced PH reached conflicting results [12,27,51]. For example, Adesina et al. [12] discovered that 72-hour in vitro hypoxia increases mtROS in human PAECs. In addition, they found that removing mitochondrial superoxide by overexpressing SOD2 exacerbates PH in CH mice, whereas scavenging mitochondrial H_2_O_2_ by overexpressing mitochondrial catalase attenuates PH, suggesting a prominent role for mitochondrial H_2_O_2_. In contrast, exposure of murine PASMCs to 5 days of hypoxia in vitro decreased mtROS levels [27]. This same group also reported that O_2_^−^ concentrations in lung homogenates are unaltered by CH in vivo and that the chronic administration of the mitochondria-targeted antioxidant MitoQ is without effect on CH-induced increases in peak RVSP. A follow-up study utilizing mice that express *Ciona intestinalis* alternative oxidase (AOX) to restore electron flux through the ETC and inhibit acute hypoxia-induced mtROS formation reached a similar conclusion [51]. While facilitating the proliferation of PASMCs in response to prolonged hypoxia in vitro, they found that AOX expression was without effect on the development of CH-induced PH.

Reports from our laboratory signify a crucial role of mtROS signaling in enhanced vasoconstrictor reactivity in a rat model of sleep apnea [28].

The protective effect of MitoQ to limit the severity of CH-induced PH in adult rats in the current study is attributed to the inhibition of CH-induced increases in pulmonary vascular resistance since cardiac output was not altered by either CH or MitoQ. This interpretation is in agreement with our findings that mtROS contribute to elevated basal PA tone and enhanced vasoconstrictor sensitivity to ET-1 after CH (Figure 5 and Figure 6A).

MtROS also appear to be central to the pulmonary arterial remodeling response to CH (Figure 4), as indicated by the attenuation of CH-induced medial layer thickening by MitoQ. ROS have further been shown to contribute to pulmonary fibrosis triggered by bleomycin [52,53,54] and asbestos [54,55]. However, whether mtROS contribute to the previously observed adventitial and perivascular fibrosis in the CH rat model [2] remains to be determined. Whereas the superoxide scavenger TEMPOL prevented the pulmonary hypertensive response to CH, it was without effect on the adventitial remodeling [2], suggesting superoxide alone does not contribute to this response. Taken together, our data strongly support the involvement of mtROS in the pathogenesis of CH-induced PH. The reason for the discrepant findings between the current study and previous work utilizing MitoQ in CH mice [27] is not clear, but it may reflect a species difference.

Whether MitoQ similarly protects against left or right ventricular dysfunction and associated myocardial cell hypertrophy, fibrosis, and inflammatory cell infiltration remains to be determined. Despite unaltered cardiac output following CH, chronic alveolar hypoxia has been documented to impair left ventricular diastolic function in mice [56]. However, limited data are available regarding the effect of MitoQ on cardiac function. Ribeiro Junior and colleagues [57] discovered that MitoQ does not improve cardiac function in a pressure overload-induced rat model of heart failure, while Supinski et al. reported that MitoQ is protective against sepsis-associated decreases in cardiac pressure generation [29]. Whether MitoQ is protective against cardiac dysfunction associated with PH represents an interesting avenue of future investigation.

The mitochondrial ETC includes four protein complexes located in the mitochondrial inner membrane and is an important source of ROS that mediates responses to both acute hypoxia [58] and ischemia–reperfusion [22]. Electrons flowing through the ETC allow for ATP production in the presence of oxygen. Normally, electrons delivered by NADH and FADH_2_ are donated to O_2_ at complex IV to form H_2_O. However, because of “electron leak” [59], a small amount of ROS are inevitably produced during this process. Complex I and complex III are major sites of ROS production within ETC, with superoxide being the primary product [59]. Chronic hypoxia has been reported to increase mtROS in both PAECs [12] and PASMCs [20]. Consistent with these findings, we observed that primary PASMCs and acutely isolated PAECs from rats exposed to CH have higher mtROS levels compared to cells from control animals (Figure 6 and Figure 7).

The contribution of mtROS to CH-induced basal arterial tone was evaluated using the pharmacologically distinct antioxidants MitoQ and MitoTEMPO, with the former preventing mitochondrial superoxide production and the latter converting mitochondrial O_2_^−^ into H_2_O_2_ [60]. Our findings that both MitoQ and MitoTEMPO effectively normalized CH-induced increases in basal tone (Figure 6A,B) in endothelium-intact PAs to control levels support the idea of a role for mitochondrial O_2_^−^, rather than mitochondrial H_2_O_2_, in this response. This finding is consistent with evidence documenting a detrimental role of O_2_^−^ in the pathogenesis of PH [2,6,16,44].

The role of MitoQ in preventing both CH-induced basal tone in endothelium-disrupted arteries and increased mtROS production in PASMCs from CH rats suggests that the coupling of mtROS to contraction is an inherent property of PASMCs. However, we additionally discovered that the endothelium exerts a mtROS-dependent vasoconstrictor influence following CH. This was revealed by dramatic increases in mtROS-mediated basal tone following the acute inhibition of NOS isoforms with L-NNA (Figure 5) to eliminate the basal vasodilatory influence of NO. The most likely source of NO that limits this endothelium-dependent vasoconstrictor response is eNOS, the primary NOS isoform involved in vasoregulation in the hypertensive pulmonary circulation. Indeed, we previously demonstrated that CH increases the expression of eNOS [61] but not iNOS [62] in this model. In addition, functional studies revealed that eNOS contributes to enhanced endothelium-dependent vasodilation [63] and attenuates vasoconstrictor reactivity [62] in the lungs of CH rats, while iNOS is without effect on these responses [62]. Although endothelial sheets from CH rats displayed greater levels of mtROS compared to cells from control animals, a role for endothelial mtROS in mediating enhanced basal tone appears unlikely when considering that L-NNA also prevented the CH-induced increase in PAEC mtROS levels. The mechanism by which L-NNA interfered with endothelial mtROS generation is not clear, but it could potentially be explained by the inhibition of mitochondrial electron transport by NO [64,65].

The ability of endothelium to exert an mtROS-dependent vasoconstrictor influence in CH arteries following NOS inhibition could alternatively be explained by endothelial potentiation of PASMC mtROS production. A possible role for the endothelium-derived, hypoxia-inducible vasoconstrictor peptide ET-1 in this response is supported by several lines of evidence. For example, NO suppresses ET-1 release from primary cultures of human umbilical vein endothelial cells under both normoxic and hypoxic conditions [66]. Additionally, eNOS inhibition by L-NNA triggers ET-1-mediated vasoconstriction in coronary arteries [67], as well as in isolated perfused lungs from CH rats [68].

Extensive evidence from our laboratory and others indicates a pathogenic role of endogenous ET-1 in both animal models of CH-induced pulmonary hypertension [7,40] and in PAH patients [8,41]. ET_R_ antagonists (i.e., bosentan, macitentan, and ambrisentan) have been approved for treating pulmonary arterial hypertension (Group I) based, in part, on early studies that found that such antagonists attenuate and reverse CH-induced PH (Group III) in rats [42]. Previous studies from our laboratory reported elevated ET-1 peptide levels in the lungs of CH rats [69]. Consistent with our findings, Kim and colleagues demonstrated that CH increased ET-1 levels in plasma from mice [37]. Furthermore, we found that ET-1 acts through ETRs to attenuate NO-dependent vasodilation [70] and augment constriction to exogenous ET-1 in endothelium-intact small pulmonary arteries from CH rats [70]. However, the role of ETRs in mediating basal tone following CH had not been previously addressed. In the present study, we found that combined ET_A/B_ receptor blockade abolished the vasoconstrictor effect of endothelium on basal tone (Figure 8), supporting our hypothesis that endogenous ET-1 potentiates mtROS signaling in the smooth muscle to augment basal tone. Further supporting this hypothesis is evidence that CH augments mtROS generation to exogenous ET-1 in primary cultures of PASMCs (Figure 9B) and mediates mtROS-dependent vasoconstriction in endothelium-disrupted PAs (Figure 9A). Similar findings suggesting that ET-1 increases mtROS have been reported in cultured ovine PAECs [71] and in mouse atrial HL-1 myocytes [72]. Taken together, our results demonstrate a previously undescribed role for endogenous ET-1/smooth muscle mtROS signaling in mediating enhanced vasoconstrictor responsiveness in small PAs after CH.

Future studies are needed to determine the mechanism by which CH increases basal and ET-1-induced mtROS production in PASMCs. Based on evidence that increases in mitochondrial [Ca^2+^] are required for mtROS production in mouse microvascular pulmonary endothelial cells [73] and in zebrafish lateral line hair cells [74], this effect of CH may result from greater mitochondrial Ca^2+^ uptake under resting conditions or secondary to ET-1-induced increases in PASMC [Ca^2+^]_i_ [75]. Also unknown is the signaling relationship between mtROS and the EGFR/NOX2 signaling pathway that we have previously implicated in mediating enhanced vasoconstrictor sensitivity following CH [20]. Considering evidence that NOX2-derived O_2_^−^ can facilitate mitochondrial Ca^2+^ uptake, as well as mtROS generation in the vasculature [24,76,77], a goal of future studies will be to examine the role of NOX2 in determining mitochondrial Ca^2+^ load, mtROS generation, and the significance of this mechanism to enhanced vasoconstrictor reactivity after CH exposure. Finally, it will be important to determine the mechanism by which mtROS lead to vasoconstriction in the hypertensive pulmonary circulation, including whether mtROS signal proximal or distal to ROK-induced myofilament Ca^2+^ sensitization [6], which we previously implicated in this response.

While experimental strategies of reducing ROS by scavenging or limiting production have shown promising outcomes in animal models of CH-induced PH [2,12,13,14,15,16], the data from humans are limited. Evidence that oxidative stress is increased in high-altitude residents [17] and in pulmonary hypertensive patients [18] justify investigations related to the effects of antioxidants on hemodynamics in patients. The possible effect of MitoQ on human PH is supported by a small-scale clinical trial studying the chemically similar compound coenzyme Q (CoQ), in which oral CoQ administration was found to improve right heart function, as assessed via echocardiography in patients with PAH [19].

In conclusion, this study supports a critical contribution of mtROS to augmented vasoconstrictor reactivity, arterial remodeling, and PH in response to CH. The ability for CH to increase spontaneous PA tone and enhance vasoconstrictor sensitivity to ET-1 is dependent on a mechanism intrinsic to PASMCs and potentiated by endothelium-derived ET-1. This study additionally represents an important step towards the development of new therapeutic strategies for the treatment of CH-associated PH that target mtROS, the translational significance of which is supported by the efficacy and safety of inhibitors of mtROS in clinical trials [78]. Challenges of future studies are to determine mechanisms by which CH increases mtROS production, potential crosstalk between NOX2 and mtROS, and how mtROS mediate the contraction of PASMCs.

## Figures and Tables

**Table 1:** Serial measurements of cardiac output and heart rate (HR) by echocardiography.

Measurement	Time of Exposure	Control		CH	
		Vehicle	MitoQ	Vehicle	MitoQ
Cardiac output (mL/min/Kg)	Baseline	277 ± 17	280 ± 27	275 ± 11	276 ± 24
	2 weeks	263 ± 11	266 ± 15	271 ± 15	266 ± 10
	4 weeks	269 ± 16	270 ± 15	262 ± 20	260 ± 8
HR (bpm)	Baseline	391 ± 4	387 ± 5	391 ± 12	389 ± 5
	2 weeks	364 ± 5	370 ± 8	379 ± 10	369 ± 6
	4 weeks	374 ± 11	371 ± 11	380 ± 7	384 ± 7

Values are mean ± SEM, *n* = 7–8 animals/group. Data were obtained in isoflurane-anesthetized rats. There are no significant differences analyzed using two-way ANOVA, followed by Tukey’s post hoc test. bpm, beats per minute.

**Table 2:** Mean systemic arterial pressure (MSAP) and heart rate (HR) measured by indwelling catheters at conclusion of 4-week exposure period.

Measurement	Control		CH	
	Vehicle	MitoQ	Vehicle	MitoQ
MSAP (mmHg)	113 ± 2	107 ± 2	117 ± 6	124 ± 2 *
HR (bpm)	362 ± 6	348 ± 14	350 ± 9	360 ± 8

Values are mean ± SEM, *n* = 5–6 animals/group. Data were obtained from isoflurane-anesthetized rats. * *p* < 0.05 vs. Control MitoQ; analyzed using two-way ANOVA, followed by Tukey’s post hoc test. bpm, beats per minute.

## Data Availability

Data are contained within the article.
